# From Re-Emergence to Hyperendemicity: The Natural History of the Dengue Epidemic in Brazil

**DOI:** 10.1371/journal.pntd.0000935

**Published:** 2011-01-04

**Authors:** Isabel Rodriguez-Barraquer, Marli T. Cordeiro, Cynthia Braga, Wayner V. de Souza, Ernesto T. Marques, Derek A. T. Cummings

**Affiliations:** 1 Department of Epidemiology, Johns Hopkins Bloomberg School of Public Health, Baltimore, Maryland, United States of America; 2 Centro de Pesquisas Aggeu Magalhães, Fundação Oswaldo Cruz, Recife, Pernambuco, Brazil; 3 Central Laboratory of Public Health, Secretaria de Saúde do Estado de Pernambuco, Recife, Pernambuco, Brazil; 4 Department of Infectious Diseases and Microbiology, Center for Vaccine Research, University of Pittsburgh, Pittsburgh, Pennsylvania, United States of America; Duke University-National University of Singapore, Singapore

## Abstract

**Background:**

Dengue virus (DENV) was reintroduced into Brazil in 1986 and by 1995 it had spread throughout the country. In 2007 the number of dengue hemorrhagic fever (DHF) cases more than doubled and a shift in the age distribution was reported. While previously the majority of DHF cases occurred among adults, in 2007 53% of cases occurred in children under 15 years old. The reasons for this shift have not been determined.

**Methods and Findings:**

Age stratified cross-sectional seroepidemiologic survey conducted in Recife, Brazil in 2006. Serostatus was determined by ELISA based detection of Dengue IgG. We estimated time-constant and time-varying forces of infection of DENV between 1986 and 2006. We used discrete-time simulation to estimate the accumulation of monotypic and multitypic immunity over time in a population previously completely susceptible to DENV. We projected the age distribution of population immunity to dengue assuming similar hazards of infection in future years. The overall prevalence of DENV IgG was 0.80 (n = 1427). The time-constant force of infection for the period was estimated to be 0.052 (95% CI 0.041, 0.063), corresponding to 5.2% of susceptible individuals becoming infected each year by each serotype. Simulations show that as time since re-emergence of dengue goes by, multitypic immunity accumulates in adults while an increasing proportion of susceptible individuals and those with monotypic immunity are among young age groups. The median age of those monotypically immune can be expected to shift from 24 years, 10 years after introduction, to 13 years, 50 years after introduction. Of those monotypically immune, the proportion under 15 years old shifts from 27% to 58%. These results are consistent with the dengue notification records from the same region since 1995.

**Interpretation:**

Assuming that persons who have been monotypically exposed are at highest risk for severe dengue, the shift towards younger patient ages observed in Brazil can be partially explained by the accumulation of multitypic immunity against DENV-1, 2, and 3 in older age groups, 22 years after the re-introduction of these viruses. Serotype specific seroepidemiologic studies are necessary to accurately estimate the serotype specific forces of infection.

## Author Summary

The spread of dengue virus is a major public health problem. Though the burden of dengue has historically been concentrated in Southeast Asian countries, Brazil has become the country that reports the largest number of cases in the world. While prior to 2007 the disease affected mostly adults, during the 2007 epidemic the number of dengue hemorrhagic fever cases more than doubled, and over 53% of cases were in children under 15 years of age. In this paper, we propose that the conditions for the shift were being set gradually since the re-introduction of dengue in 1986 and that they represent the transition from re-emergence to hyperendemicity. Using data from an age stratified seroprevalence study conducted in Recife, we estimated the force of infection (a measure of transmission intensity) between 1986–2006 and used these estimates to simulate the accumulation of immunity since the re-emergence. As the length of time that dengue has circulated increases, adults have a lower probability of remaining susceptible to primary or secondary infection and thus, cases become on average younger. If in fact the shift represents the transition from re-emergence to hyperendemicity, similar shifts are likely to be observed in the rest of Brazil, the American continent and other regions where transmission emerges.

## Introduction

Dengue infection constitutes a major threat for urban populations of Latin America and Asia [1],[2]. Important differences in the clinical and epidemiological profile of dengue between the countries of Latin America and Southeast (SE) Asian have been observed. While in SE Asian countries dengue hemorrhagic fever (DHF) is common and morbidity and mortality has traditionally concentrated in children under 15 years of age, in American countries the disease affects mostly adult populations and manifests primarily as dengue fever (DF) [3], [4].

Several hypotheses have been proposed to explain these differences. It has been shown that children from Central America, Venezuela, and Colombia may not develop vascular permeability as readily as children from SE Asia after secondary dengue infection,[5]–[6] and that there may be a high prevalence of dengue resistance genes among black populations of Brazil and the Caribbean [7], [8]. An additional explanation for the low numbers of DHF in American countries may be underreporting of cases that do occur, due to technical difficulties or a limited capacity to perform diagnosis that meet the criteria of the WHO case definition [3]. None of these explanations are fully satisfactory in explaining the differences between the two regions.

Dengue was reintroduced in Brazil in 1986, after an absence of at least 20 years (except for an epidemic in Roraima in 1981 and sporadic cases). Since then, Brazil has become the country that reports the largest number of cases to the WHO, accounting for over 70% of cases reported in the Americas [9], [10]. Three serotypes currently circulate throughout the country; DENV 1 was reintroduced to Rio de Janeiro in 1986, DENV 2 in 1990, and DENV 3 in 2002, and from Rio they spread to the rest of the country[11]. While prior to 2007 the majority of DHF cases in Brazil occurred among adults aged 20–40 years of age, in 2007 the annual number of DHF cases more than doubled over previous years and a shift in the age distribution was reported [12]. In 2007, 53% of cases occurred in children under 15 years old. The shift was most noticeable in the Northeast region, where children accounted for 65% of the total number of DHF cases, while other regions such as the Central-West and North did not experience a significant shift and most of the DHF cases continued to occur among adults [12].

Although the cause of this shift is likely to be multifactorial, we propose that the conditions for it were being set gradually since the re-emergence of DENV in 1986 and that the current epidemiological profile represents the transition from re-emergence to hyperendemicity. In a setting where transmission is constant, people who are exposed for a longer time have a greater cumulative probability of infection. In Brazil, circulation of DENV virus for over 20 years has resulted in the accumulation of immunity in older individuals, driving the average age of primary and secondary infection towards younger age groups.

Using data from a serological study performed in Recife, in Northeast Brazil, we estimate the force of infection and basic reproductive number of dengue in three areas of distinct socio-economic status for the period 1986–2006, in order to better understand transmission intensity over this period. We then use these estimates to simulate the accumulation of monotypic and multitypic immunity in a population previously susceptible to dengue virus, and to predict the expected age distribution of DHF cases in the future.

## Materials and Methods

### Ethics statement

This study was reviewed and approved by the ethics committee of the CPqAM-Fiocruz/Brazilian Ministry of Health (No. 49/04). Written consent to participate in the study was obtained from each person (or their guardian) after a full explanation of the study was provided. All personal identifiers were removed prior to secondary data analysis at Johns Hopkins University.

### Data sources

This study was based on a serological sample of households in Recife, Pernambuco, Brazil, conducted between August and September 2006. The first dengue outbreak in the state of Pernambuco occurred in 1987 (DENV 1). No additional autochthonous cases were reported until 1995, when DENV 2 was introduced causing a new epidemic. Since 1995 cases have been reported every year. DENV 3 was first isolated in Pernambuco in 2002 [13].

The study population and methods have been described in detail by Braga et al. [14] Briefly, Recife has 1.5 million inhabitants. The climate is humid, with an average temperature of 25°C and rainfall of approximately 2000 mm per year. Three neighborhoods were selected to represent low, medium and high socio-economic areas. A systematic age stratified sample was obtained, using the Census 2000 data that provides the total population size, number of households and age distribution in the three areas [15]. Residents aged between 5 and 64 years were eligible for the survey. Serum samples were screened for IgG antibodies against DENV with an enzyme-linked immunoassay commercial kit (Dengue IgG-ELISA, PanBio, Ltd., Brisbane, Australia). Tests were performed in duplicate according to the manufacturer's instructions. This test does not determine the presence of immunity to specific dengue serotypes, but the presence of immunity to any dengue serotype.

### Estimating the force of infection

The force of infection (λ) is a measure used to characterize the intensity of transmission in a given setting and estimates the per capita rate of acquisition of infection by susceptible individuals. Age stratified serological surveys can provide information about the force of infection over a period of time, λ(t), as described elsewhere [16]. Assuming that the risk of infection does not vary with age, the difference in seroprevalence between subjects *a* and *a+1* years of age can be attributed to the transmission intensity between *a* and *a+1* years ago. To estimate λ(t), for the period 1986–2006, we used a model based upon one described by Ferguson et al.[17] We estimated constant and time-varying forces of infection. Detailed information regarding the methods used can be found in Text S1 in Supporting Information S1.

### Estimating the basic reproductive number (R_0_)

R_0_ is the number of secondary infections generated by a primary case in a completely susceptible population. R_0_ gives insight into the level of control that is required to reduce incidence and eventually block transmission. Detailed information regarding the methods used to estimate R_0_ can be found in Text S1 in Supporting Information S1.

### Immunologic shift simulations

To estimate the accumulation of monotypic and multitypic immunity in a population previously susceptible to dengue, we performed a discrete-time simulation by applying the forces of infection estimated from the seroprevalence data onto a simulated immunologically naive population structured by-age like the one of Recife. We used independent data on the years in which the different serotypes were introduced into Brazil/Pernambuco to apply the estimated hazards only in those years when particular serotypes were known to have circulated [11], [13]. The age profile of the population was obtained from the 2000 census data. We conducted simulations until age distributions of immunity reached equilibrium and used both constant and time-varying hazards.

Since we did not have seroprevalence data to estimate the force of infection beyond 2006, we assumed that λ(t) after 2006 has been constant and equal to the average hazard over the period 1986–2006.

All statistical analyses were performed using R statistical package (version 2.10.1).

## Results

**Figure 1:**
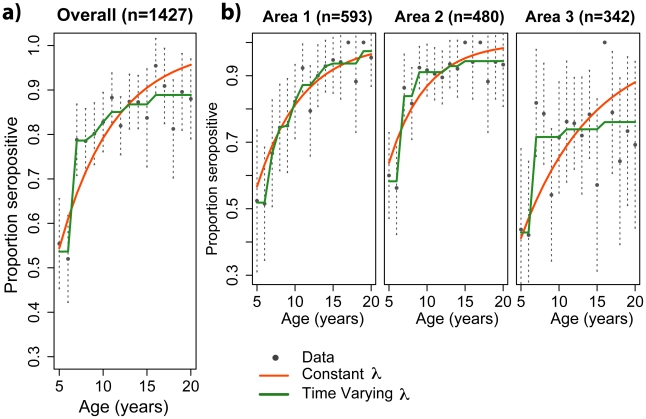
Age specific seroprevalence of dengue in Recife, 2006. Age-specific seroprevalence data a) for the whole sample and b) for each specific area. The lines show the fit of 1) constant (red lines) and 2) time-varying models (green lines) to the age-specific seroprevalence data (black dots). 95% confidence intervals of seroprevalence data are shown by the dotted vertical lines.

The dataset contained data on 1427 subjects aged 5 to 20 years, 593 (41.6%) from area 1, 480 (33.6%) from area 2 and 342 (24.0%) from area 3. Figure 1 shows the age-specific seroprevalences of each of the areas (black dots). Area 1, the neighborhood of low socioeconomic status showed a significantly higher seroprevalence when compared to Area 3, the high socioeconomic stratum neighborhood (0.85 (95%CI 0.82–0.88) vs. 0.70 (95%CI 0.65–0.75), p<0.0001). The middle class neighborhood (Area 2) also showed a significantly higher seroprevalence when compared to Area 3 (0.82 vs. 0.70, p = 0.0002). Based on the seroprevalence and that approximately 87000 cases were notified in Recife during this period, it is clear that less then 10% of the infections were reported [13].

### Force of infection

The estimated average time-constant force of infection for the period 1986–2006 was 0.052 (95% CI 0.041–0.063). On average, each serotype infected 5.2% of susceptible individuals each year.

Time constant 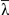
s for the three areas were 0.068 (95%CI 0.045, 0.091), 0.056 (95%CI 0.035, 0.077) and 0.035 (95%CI 0.019, 0.051). Though the difference between these forces of infection is not statistically significant, a trend is seen towards higher hazards of infection in settings of lower socioeconomic status.

**Figure 2:**
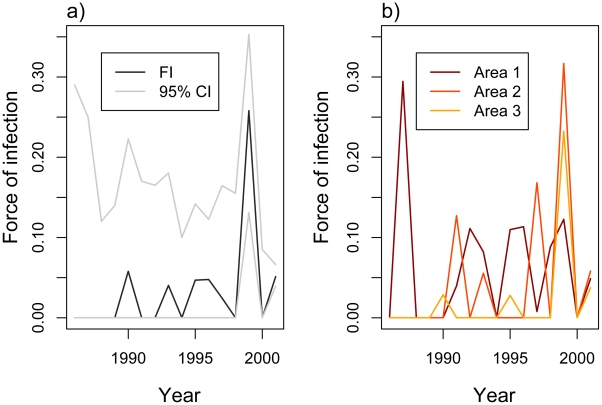
Estimated average time-varying forces of infection. a) Estimated average time-varying forces of infection (FI), with 95% confidence intervals for Recife over the period 1986–2006. b). Estimated time varying-forces of infection for the three sampled neighborhoods over the period 1986–2001.

As can be expected, the fit of the model improved significantly when we allowed for time-varying forces of infection (likelihood ratio test, p = 0.006). According to this model (Figure 2), the average yearly force of infection ranged between 0 and 0.057 between 1986 and 1998, and then peaked at 0.26 in 1999. As has been reported elsewhere the correlation between incidence and estimated force of infection is poor (r = 0.21) [18].

Given that it has been reported that between 1987 and 1995 there were no autochthonous dengue cases in the state of Pernambuco, we also fit a model constraining the force of infection for these years to be 0 [13]. The fit of this 8-parameter model was not significantly different from the fit of the model that did not constrain these hazards to be zero (LR test, p = 0.99) or from the saturated model (LR test, p = 0.34).

Figure 1 shows the fit of 1) constant (red lines) and 2) time-varying models (green lines) to the age-specific seroprevalence data in the three areas and overall areas.

The correlation between the annual hazards estimated in areas 1 and 3 (r = 0.79) is high, while the correlation between 1 and 2 and between 2 and 3 is poor (r = 0.06 and 0.18, respectively).

### Basic reproductive number

Using the time constant and time varying *λ*s we estimated an overall R_0_ of dengue in Recife of 2.7 (95%CI 2.45, 3.11). For the three areas the R_0_ estimates were 3.3 (95%CI 2.45, 4.18), 2.8 (95%CI 2.09, 3.64) and 2.1 (95%CI 1.56, 2.66), respectively.

### Simulations

**Figure 3:**
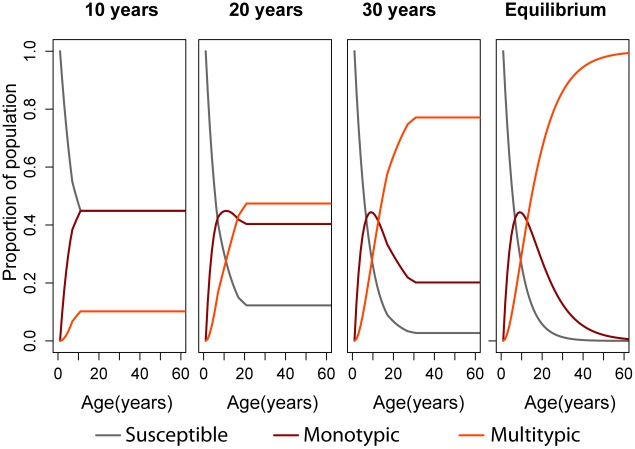
Discrete time simulation shows that as years go by, multitypic immunity accumulates among adults. Results of discrete-time simulation to show the accumulation of multitypic and monotypic immunity 10, 20, and 30 years after the introduction of dengue virus into a previously susceptible population, and at equilibrium. 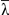 (DENV 1, 2 and 3)  = 0.05.

Figure 3 shows the age distribution of susceptible, monotypically immune and multitypically immune at different time-points after the introduction of DENV 1, 2 and 3 into a previously susceptible population, assuming a constant risk of infection of 0.052/year/serotype. As the number of years of DENV circulation increases, multitypic immunity accumulates among adults, and susceptibles and monotypically immune become increasingly concentrated in younger age groups.

**Figure 4:**
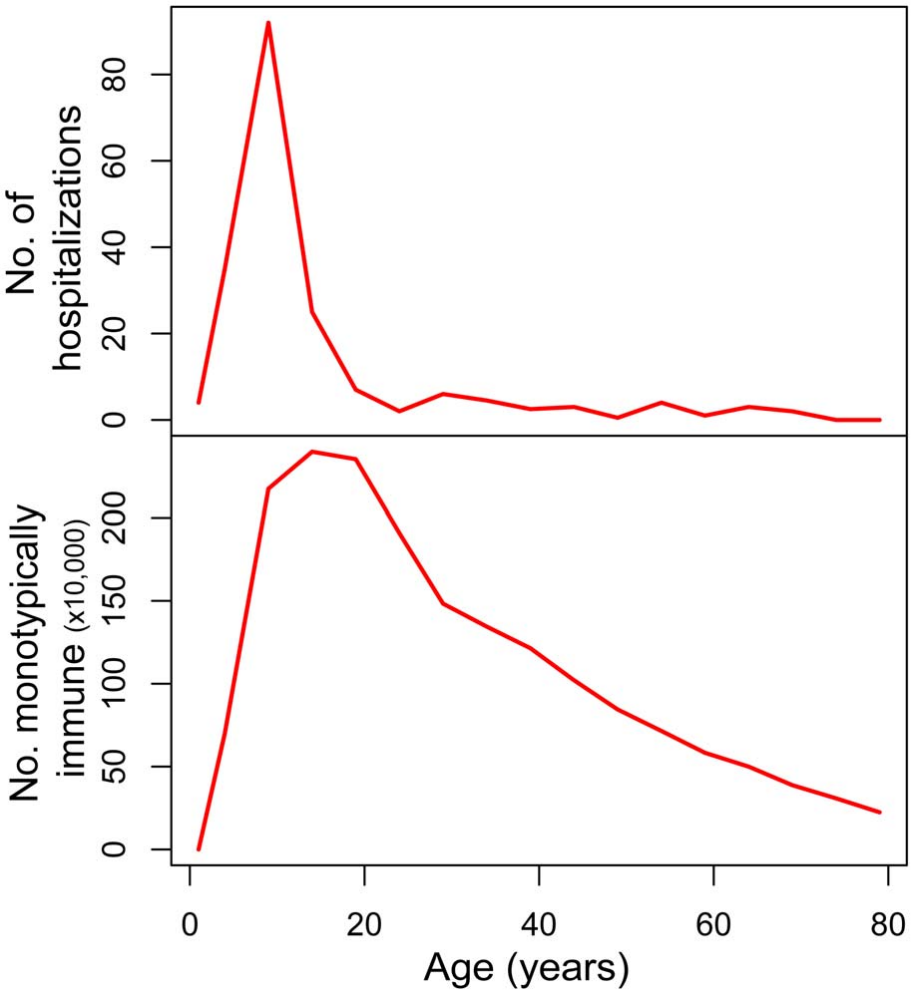
Age distribution of hospitalized dengue cases in Brazil, 2007 resembles expected age distribution of monotypically immune. Age distribution of hospitalized dengue cases in Pernambuco, 2007 [20] (top). Age distribution of monotypically immune, 20 years after the introduction of dengue virus into a previously susceptible population, as predicted by the model (bottom). λ (DENV 1, 2 and 3)  = 0.05.

Assuming that cases of DHF occur primarily among people who experience secondary infection, the age distribution of people who are at risk of secondary infection (i.e. of people who have been exposed to a single dengue serotype) should approximate the age distribution of DHF cases [19]. Hence, our results suggest that as years after re-emergence go by, the mean, median and modal ages of cases will decrease. For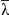  = 0.052, the model estimates that while 10 years after re-emergence the median, mean and modal age of cases (monotypically immune) would be 24, 29.0 and 14 years respectively, these numbers would decrease to 13, 15.2 and 11 years 50 years after re-emergence. Similarly, while it is expected that only up to 27% of DHF cases would occur in children under 15 years of age 10 years after the re-emergence, 50 years after re-emergence this proportion would increase to 58%. Figure 4 shows the age distribution of hospitalized dengue cases in Pernambuco in 2007, based on official notification records, and the estimated distribution according to our model (20 years after re-emergence) [20].

**Figure 5:**
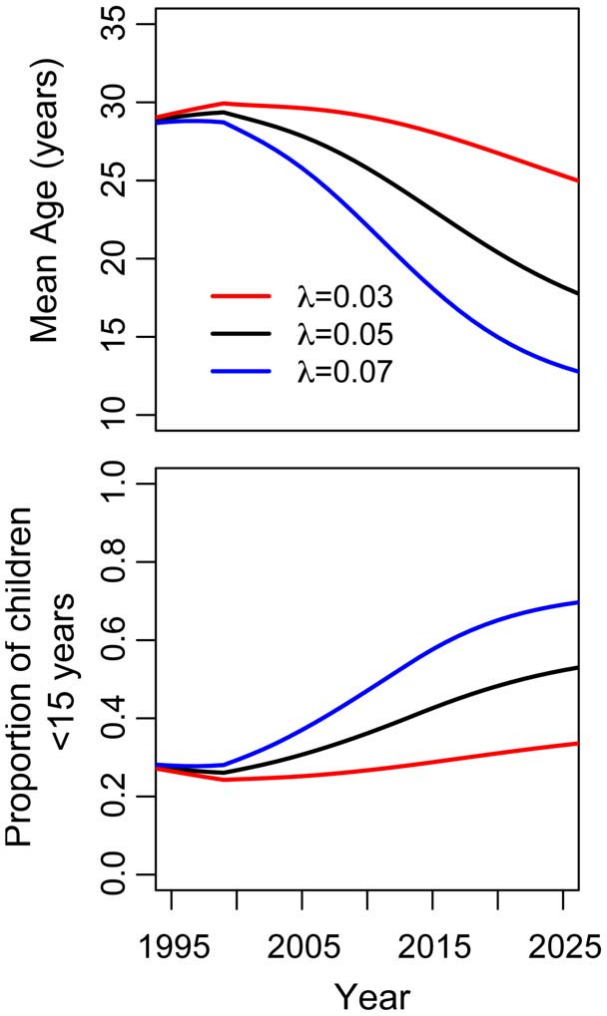
Predicted changes in the age distribution of monotypically immune (DHF/DSS) over time. Estimated mean age of monotypically immune (top) and proportion of monotypically immune (DHF/DSS cases) that are children under 15 years (bottom) at several time points after the introduction of DENV into a previously susceptible population.

The strength and the speed of the shift in the age distribution of immunity depend on the underlying force of infection (Figure 5 and Table S1 in Supporting Information S1). The estimated median and modal ages of monotypically immune for 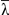 = 0.03 are 19 and 15 years respectively, 50 years after re-emergence, while these ages drop to 11 and 6 years for 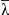 = 0.07.

Results were similar if time-varying, instead of constant forces of infection were applied, or if 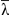 for each serotype was weighted taking into account the serotype predominance reported for the different years in the state of Pernambuco [13].

## Discussion

A dramatic increase in the number of DHF cases and a shift in age group predominance of DHF were observed during the 2007 dengue epidemic in Brazil, the first re-emergence of the DENV-2 serotype predominance since 1990. Our results suggest that this shift can be partly explained by the accumulation of multitypic immunity in the adult population over time after the re-emergence of DENV-1 in 1986, DENV-2 in 1990 and DENV-3 in 2002. As the length of time of co-circulation of multiple serotypes of dengue in Brazil increases, adults have a lower probability of remaining susceptible to infection. As a result, cases become on average younger as completely susceptible individuals and monotypically immune individuals are more likely to be from younger age groups.

If the accumulation of multitypic immunity in adult population is in part responsible for the observed shift in age group predominance of severe dengue cases, we would expect similar shifts to have occurred in central and northern South America, where several DENV serotypes have been known to circulate since the 1970's. In Mexico, Venezuela, Nicaragua and Colombia most of the severe cases occur among children <15 years of age and a similar trend is being observed in Honduras.[21], [22],[23]. In contrast, such a trend has not been observed in countries where multiple serotypes only started circulating in the 90's. If the central/west regions of Brazil continue to experience high DENV forces of infection and multiple circulating serotypes we expect a similar shift in age group predominance to occur in the coming years.

Since DHF is more likely to occur in children, a decrease in the mean age of secondary infection might also be expected to lead to an increase in the proportion of dengue infections that lead to severe symptoms or DHF cases [24], [25]. In the 1990's, after DENV-2 was introduced, 0.06% of reported dengue cases in Brazil resulted in DHF/DSS. This percentage increased to 0.21% in 2007 [9]. This observed increase in DHF may also have been a result of changes in virulence of particular dengue viruses that were circulating or due to the fact that the overall force of infection has increased as has been proposed.

According to our model, the speed of the shift is proportional to the magnitude of the average force of infection. Higher average forces of infection lead to a more rapid shift of the age distributions of immunity and to a younger median and modal age of monotypically immune. Thus, the shift can be expected to be slower in regions that have been exposed to weaker forces of infection or where the re-emergence of multiple serotypes was delayed. This may explain why the shift has only been observed in major cities and certain regions of Brazil. The Northeast region of Brazil, where Recife is located, has the highest proportion of children among DHF cases, and it has also been traditionally the region with the highest incidence rates of dengue fever since 1986 [9], [12]. The Central-West region, where the shift is not yet apparent has shown high incidence rates of dengue fever only during the last 7 years [9].

Our estimate of the average λ and R_0_ in Recife is lower than those estimated for Thailand for the period 1980–2005 (λ = 0.1, R_0_ = 5.2) [26]. Our model predicts that average forces of infection of 0.1 would be associated with a mean age of severe or DHF cases of 8 years, and this is consistent with what has been traditionally observed in SE Asian countries. Both Thailand and Singapore have experienced significant decreases in transmission intensity over the last few years that have been accompanied by an increase in the average age of cases [26],[27],[28]. If the force of infection in Recife continues to be as high as it has been over the last 20 years, or higher, it is likely that within the next decade the age distribution of DHF in Recife (and other American regions with high forces of infection) will resemble the age distribution observed in SE Asia, with most cases concentrated in the adolescent population. However, our projections are meant to be qualitative rather than quantitative. The actual seroprevalences observed in the future in Recife may differ from our projections depending on secular trends in the transmission intensity of dengue and population demographics.

There are several limitations to this study. Even though our results present an explanation for why DHF may have shifted towards children over the years since introduction, the mechanism that we propose is gradual and does not explain the sudden change observed in 2007–2008. The recirculation of DENV-2 into certain cities in 2007, after almost 7 years of DENV 3 predominance and the resultant increase in secondary cases may have determined the observation of an age shift in 2007 and not before, even though it had been gradually taking place [9]. As reported by the Ministry of Health, during 1998–2006 the percentage of severe dengue cases in children increased from 9.5% (in 1998) to 22.6% (in 2001). Although our results suggest that the major driver of the shift is the accumulation of immunity in older age groups, fluctuations in serotype specific transmission intensity, serotype predominance, characteristics of the virus or serotype predominance may have also played a role in determining the visibility of the shift.

Our model predicts that after 20 years of exposure to a constant force of infection of 0.05 per year, children 15 years old or younger should only account for 31% of DHF cases while the data shows that in 2007, 70% of cases in Recife occurred among children of this age group. This discrepancy may arise due to the fact that the model does not take into account age-dependence of infection or clinical presentation. If children are more likely to develop severe disease, then the observed distribution of cases is likely to be skewed towards lower age groups.

The fact that the available serological study does not contain serotype specific information limits our ability to estimate serotype specific forces of infection, interactions (enhancement/inhibition) and basic reproductive numbers. Similarly, the cross-sectional nature of this dataset does not allow us to control for potential confounding by age dependent transmission intensity. Longitudinal data and data from seroprevalence studies using serotype specific methods such as the PRNT are essential in order to properly reconstruct the transmission intensity over the last 20 years.

This analysis has important public health implications on planning public health responses to dengue for the next decade. Dengue is the most rapidly spreading vector borne viral disease. If the age shift in fact represents the transition from re-emergence to hyperendemicity, similar shifts in age are likely to be observed in the rest of Brazil, the American continent and other regions where dengue has emerged more recently.

## Supporting Information

Supporting Information S1 Detailed methods and additional results.
(0.07 MB DOC)
