# Supplementary material for: From Re-Emergence to Hyperendemicity: The Natural History of the Dengue Epidemic in Brazil
Source: PLoS Negl Trop Dis. 2011 Jan 4;5(1):e935. doi: 10.1371/journal.pntd.0000935 (PMC3014978; doi:10.1371/journal.pntd.0000935)
Supplement: Supporting Information S1 — Detailed methods and additional results. (0.07 MB DOC) [file pntd.0000935.s001.doc]

**Supporting Information S1**

**Text S1**

Estimating the force of infection

The force of infection (λ(t)) is a measure used to characterize the intensity of transmission in a given setting and estimates the per capita rate of acquisition of infection by susceptible individuals. Age stratified serological surveys can provide information about the force of infection over a period of time, as described elsewhere [1]. Assuming that the risk of infection does not vary with age, the difference in seroprevalence between subjects *a* and *a+1* years of age can be attributed to the transmission intensity between *a* and *a+1* years ago. To estimate λ(t), for the period 1986-2006, we used a model based upon one described by Ferguson et al.[2]

The proportion of susceptibles aged *a* at time *t* (*x(a,t))* can be expressed as

(1)

where λ_k_ represents the force of infection of serotype k.

Similarly, assuming non-interacting serotypes, the proportion of individuals monotypically infected by serotype i *(z_i_(a,t))* can be expressed as

(2)

Given that ELISA IgG does not differentiate between serotypes of DENV or between monotypic or multitypic immunity, we can only estimate a force of infection, *λ(t) ,*  which represents a weighted average of all serotypes that are circulating in Brazil,*.*

The multinomial log-likelihood of the data reduces to a binomial log-likelihood where the two outcomes are escaping infection or experiencing one or more infections. This binomial log-likelihood is given by,

(3)

The parameter *λ*(t) is then estimated by maximizing equation 3 using non-linear optimization methods. The goodness of fit of the models can be assessed via a likelihood ratio test, comparing more complex to simpler models and by comparing the maximum log-likelihood (l_max_) estimate to the saturated log likelihood (l_sat_). We approximated 95% confidence boundaries using conditional likelihood methods, as the range of values of the parameter for which the log-likelihood is within ½ χ^2^ of the maximum log-likelihood.

We estimated 1) constant (average) forces of infection (1 parameter model) for the period 1986-2006 and 2) time-varying forces of infection for the period 1986-2001, assuming constant hazard across age groups but a specific *λ*(t) for each year 1986 to 2001 (16 parameter model).

We estimated distinct constant and time-varying forces of infection for the each of three areas where the samples were obtained.

Estimating the basic reproductive number (R_0_)

R_0_ is the number of secondary infections generated by a primary case in a completely susceptible population and is critical because it gives insight into the level of control that is required to reduce incidence and eventually block transmission.

Using the time constant and time varying forces of infection, we estimated R_0_ of dengue overall and for each area, as the inverse of the proportion of the population that remains susceptible, using the age structure of the population reported in the 2000 Census for Recife.

(4)

where *f(a)* is the proportion of the population of age a and *w(a,t)* is the proportion of people aged *a* at time *t* who remain unexposed to each serotype. Since *w* can’t be estimated directly (given the lack of specificity of serologic methods), it has to be estimated from *λ(t)* as

(5)

Given that dengue was reintroduced into Brazil in 1986 and that the youngest age strata in the data set were children 5 years old, we only had λ estimates for a period of 16 years. In order to estimate R_0_ for the entire population, we assumed that λ from 2006 onwards was constant and equal to the average λs for the period 1986-2006.

References

[1] Grenfell BT, Anderson RM. The estimation of age-related rates of infection from case notifications and serological data. J Hyg (Lond). 1985 Oct;95(2):419-36.

[2] Ferguson NM, Donnelly CA, Anderson RM. Transmission dynamics and epidemiology of dengue: insights from age-stratified sero-prevalence surveys. Philos Trans R Soc Lond B Biol Sci. 1999 Apr 29;354(1384):757-68.

**Table S1 :**  Results of simulation after applying different constant forces of infection ****λ(t) to a susceptible population for different amounts of time· Median age, modal age and proportion < 15 years of monotypically immune.

| **Hazard *λ(t)*** | **0.03** | | | | **0.052** | | | | **0.07** | | | |
| --- | --- | --- | --- | --- | --- | --- | --- | --- | --- | --- | --- | --- |
| Time since introduction (years) | 10 | 20 | 30 | 50 | 10 | 20 | 30 | 50 | 10 | 20 | 30 | 50 |
| Proportion of monotypically immune <15 years | 0.26 | 0.25 | 0.29 | 0.36 | 0.27 | 0.31 | 0.43 | 0.58 | 0.28 | 0.37 | 0.58 | 0.72 |
| Median age | 25 | 25 | 23 | 18 | 24 | 23 | 17 | 13 | 24 | 20 | 12 | 10 |
| Modal age | 14 | 17 | 14 | 14 | 14 | 12 | 11 | 11 | 14 | 5 | 5 | 5 |
